# Higher plasma neurofilament-light chain concentration in drug-resistant epilepsy

**DOI:** 10.1093/braincomms/fcaf108

**Published:** 2025-03-11

**Authors:** Sarah Akel, Markus Axelsson, Fredrik Asztely, Henrik Zetterberg, Johan Zelano

**Affiliations:** Department of Clinical Neuroscience, Institute of Neuroscience and Physiology, Sahlgrenska Academy, University of Gothenburg, Gothenburg 41345, Sweden; Wallenberg Centre for Molecular and Translational Medicine, Gothenburg 41126, Sweden; Department of Clinical Neuroscience, Institute of Neuroscience and Physiology, Sahlgrenska Academy, University of Gothenburg, Gothenburg 41345, Sweden; Department of Neurology, Sahlgrenska University Hospital, Member of ERN Epicare, Gothenburg 41345, Sweden; Department of Clinical Neuroscience, Institute of Neuroscience and Physiology, Sahlgrenska Academy, University of Gothenburg, Gothenburg 41345, Sweden; Department of Neurology, Angered Hospital, Angered 42422, Sweden; Department of Psychiatry and Neurochemistry, Institute of Neuroscience and Physiology, Sahlgrenska Academy, University of Gothenburg, Mölndal 43180, Sweden; Clinical Neurochemistry Laboratory, Sahlgrenska University Hospital, Mölndal 43180, Sweden; Department of Neurodegenerative Disease, UCL Institute of Neurology, London WC1E 6BT, UK; UK Dementia Research Institute at UCL, London WC1E 6BT, UK; Hong Kong Center for Neurodegenerative Diseases, Hong Kong 1512-1518, China; Wisconsin Alzheimer's Disease Research Center, University of Wisconsin School of Medicine and Public Health, University of Wisconsin–Madison, Madison, WI 53726, USA; Department of Clinical Neuroscience, Institute of Neuroscience and Physiology, Sahlgrenska Academy, University of Gothenburg, Gothenburg 41345, Sweden; Wallenberg Centre for Molecular and Translational Medicine, Gothenburg 41126, Sweden; Department of Neurology, Sahlgrenska University Hospital, Member of ERN Epicare, Gothenburg 41345, Sweden

**Keywords:** blood-based biomarkers, brain injury, drug-resistant epilepsy, neurodegeneration

## Abstract

Drug-resistant epilepsy is the most severe form of epilepsy and is frequently associated with cognitive decline. Whether drug-resistant epilepsy results in neurodegeneration or other types of brain injury is not known, and early detection of detrimental clinical trajectories would be clinically very useful. Blood biomarkers of brain injury reflect neurodegeneration or brain injury in several brain diseases but have not been extensively studied in epilepsy. We investigated a panel of such markers in a large epilepsy cohort with an emphasis on assessing differences between drug-resistant and monotherapy-controlled epilepsy. Blood neurofilament light, glial fibrillary acidic protein, total tau, S100 calcium-binding protein B and neuron-specific enolase concentrations were measured in 444 patients (aged ≥ 18 years) with epilepsy participating in a prospective regional Biobank study in Västra Götaland (Sweden). Multiple linear regression assessed associations between clinical variables and marker levels. Levels were then compared between patients with drug-resistant epilepsy (*n* = 101) and patients with monotherapy-controlled epilepsy (*n* = 164). We also performed logistic regression analysis to evaluate the significance of the markers as predictors of epilepsy status (drug-resistant epilepsy or monotherapy-controlled epilepsy) while controlling for clinical variables: age, sex, epilepsy duration, epilepsy type and lesions. All markers correlated with age. In younger patients (≤50 years), cases of drug-resistant epilepsy had higher levels of neurofilament light (*P* = 0.002) and glial fibrillary acidic protein (*P* = 0.006) compared with monotherapy-controlled epilepsy. After excluding patients with known structural lesions, neurofilament light levels remained significantly elevated in drug-resistant epilepsy versus monotherapy-controlled epilepsy (*P* = 0.029). Neurofilament light also emerged as a significant predictor of drug-resistant status in a logistic regression model following adjustments for clinical variables. Future studies should explore if neurofilament light can be used for surveillance of disease course and whether it reflects brain injury in drug-resistant epilepsy.

## Teaser

Individuals with drug-resistant epilepsy may suffer from cognitive decline. Akel *et al*. examined levels of blood–brain injury biomarkers in epilepsy, with a focus on drug-resistance. They found higher levels of neurofilament light and glial fibrillary acidic protein in drug-resistant epilepsy. Neurofilament light remained significantly increased even after excluding patients with brain lesions.

## Graphical Abstract

**Graphical Abstract:**
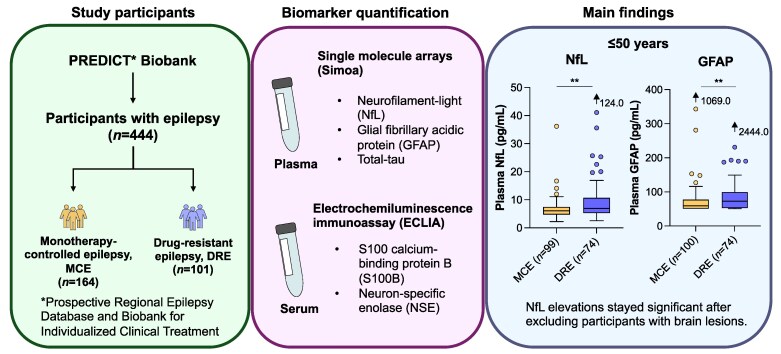

## Introduction

Epilepsy is a neurological disorder characterized by a pre-disposition for recurrent seizures, affecting ∼1–2% of the global population.^1,2^ While most patients can manage their seizures with anti-seizure medication (ASM), one-third of patients do not respond to drugs and continue to have uncontrollable seizures.^3^ Often, the most effective treatment for these patients is surgical resection of the brain tissue responsible for the seizures, though success rates can vary widely, between 20 and 80%.^4^ Patients with drug-resistant epilepsy (DRE) experience higher mortality rates, an increased risk of comorbidities and an overall reduced quality of life.^5-7^ Cognitive impairment, such as reduced executive function, is a frequent complication of DRE.^8,9^

Biochemical blood markers of brain injury are increasingly linked to cognitive decline and neurodegeneration.^10^ Plasma neurofilament light (NfL) and glial fibrillary acidic protein (GFAP) have been associated with dementia, cognitive decline and poor functional independence in daily activities.^11,12^ Similarly, elevated plasma tau is also associated with cognitive decline.^13,14^ Higher levels of the astrocytic injury marker S100 calcium-binding protein B (S100B) have been linked to cognitive dysfunction in various conditions.^15-17^

Studies on blood biomarkers for brain injury are also emerging in epilepsy. NfL seems related to seizures.^18-21^ We previously described higher plasma NfL in patients with recent seizures than in those seizure-free.^22^ Higher levels of serum total tau have been reported in adult patients with self-limiting tonic–clonic seizures.^23^ Neuron-specific enolase (NSE) has been investigated as a prognostic marker of neurological outcomes after traumatic brain injury (TBI).^24,25^ The utility of NSE as a marker in epilepsy remains uncertain, though several studies have reported increased blood levels to be associated with seizures.^26-29^ Several studies have also implicated GFAP and S100B as potential markers in epilepsy.^21,29-33^

DRE is a severe disease phenotype often associated with cognitive decline.^9^ In this cross-sectional study, we analysed plasma NfL, GFAP, total tau and serum S100B and NSE in a large regional cohort of adults with epilepsy. We compared marker levels in patients with DRE and monotherapy-controlled epilepsy (MCE) to assess if there is biochemical evidence of brain injury in DRE similar to that seen in studies on cognitive decline. If so, blood biomarkers for brain injury may show clinical utility in identifying particularly severe epilepsy trajectories.

## Materials and methods

### Cohort and clinical data collection

The Prospective Regional Epilepsy Database and Biobank for Individualized Clinical Treatment (PREDICT). PREDICT is a regional Biobank study based in Region Västra Götaland (VGR), Sweden (clinicaltrials.org, NCT04559919) which aims to identify biomarkers of epilepsy. Participants for PREDICT are recruited from five outpatient clinics in VGR starting from December 2020. The study is approved by the Ethical Review Authority (approval number 2020-00853) and followed the principles of the Declaration of Helsinki. All persons provide written informed consent before inclusion in the study. To be eligible for the study, participants have to be 18 years or older, and either have experienced an unprovoked seizure within the last year or have an epilepsy diagnosis as per the current definition by the International League Against Epilepsy (ILAE). Individuals with an expected survival of <2 years or those unable to provide informed consent are excluded. Clinical data were collected from medical records and information obtained from the recruitment appointment into a pseudonymized clinical report form by a neurologist (F.A. or J.Z.). Clinical variables used in this study were: age, sex, structural lesions, epilepsy duration, type of epilepsy and anti-seizure medications.

**Figure 1:**
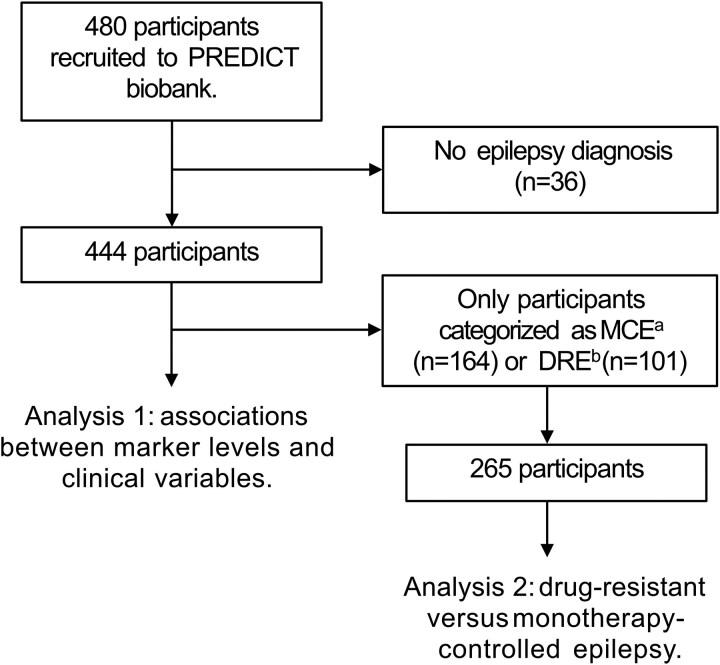
**Flow chart depicting participant selection process.**  ^a^Monotherapy-controlled epilepsy. ^b^Drug-resistant epilepsy.

As of April 2023, 480 participants had been recruited to PREDICT. For this particular study, we included all participants with an epilepsy diagnosis (*n* = 444). Treatment responsiveness was categorized into DRE, MCE and undetermined. A total of 101 individuals were categorized under DRE, 164 with MCE and the remaining 179 as undetermined (Fig. 1). DRE was defined by participants on two or more anti-seizure medications who were not seizure-free at inclusion/sampling. MCE was defined by participants with seizure freedom longer than 1 year on monotherapy only.

### Blood collection and quantification of biomarkers

After recruitment, blood samples were drawn into EDTA tubes for plasma and serum tubes for serum. The samples were then centrifuged at 2000*×g* for 10 min at room temperature. Samples were aliquoted and stored in Biobank Väst at −80°C until further analysis. Biomarker quantification was conducted at the Clinical Neurochemistry Laboratory at Sahlgrenska University Hospital (Mölndal, Sweden). Plasma NfL [picograms per millilitre (pg/mL)], GFAP (pg/mL) and total tau (pg/mL) concentrations were measured using the Single molecule array (Simoa) N4PB kit on an HD-1 Analyzer (Quanterix). The intra-assay coefficients of variation were 10% or lower.

Serum S100B [microgram per litre (µg/L)] and NSE [nanograms per millilitre (ng/mL)] concentrations were measured using the electro-chemiluminescence immunoassay (ECLIA) on the cobas e601 module (Roche Diagnostics). The methods were performed according to the manufacturers’ instructions.

### Statistical analysis

All statistical analyses were performed using SPSS (IBM SPSS Statistics for Macintosh, version 29.0.2) or GraphPad Prism (GraphPad Software, Boston, MA, USA, version 10.2.3 for Macintosh). Since the biomarkers showed a non-normal distribution, we used non-parametric tests. To analyse correlations between the markers and age, we used Spearman rank correlation (correlation coefficient expressed as Spearman’s rho = *r*_s_). We performed partial correlation analysis to explore the correlation between the markers and epilepsy duration while accounting for the potential impact of age on the marker levels. To examine the relationship between the biomarkers and the clinical characteristics epilepsy duration, days since the last seizure, structural brain lesions, epilepsy type and mono/polytherapy, we performed a multiple linear regression analysis, controlling for age and sex. For this regression analysis, the biomarkers were natural log-transformed to achieve a normal distribution of the residuals, and the regression coefficients were expressed in terms of a relative percentage change *B*_rel_ (%) = (exp(*b*) − 1) × 100%) to enhance the interpretability of the results. To compare absolute marker levels between individuals with DRE and MCE, we performed the Mann–Whitney U-test (two-sided, *P* < 0.05). The analysis was performed in two age groups, with 50 years of age as the cut-off since this is when normal values of NfL start to show a more pronounced increase, presumably because of age-related brain changes.^34^ A binary logistic regression was conducted to investigate the relationship between the outcome DRE versus MCE and marker level when controlling for age, sex and epilepsy duration. In a second set of models, we also adjusted for structural lesions and epilepsy type. A Kruskal–Wallis test (*P* < 0.05) with multiple comparisons adjustment (Benjamini, Krieger and Yekutieli method^35^) was performed to compare MCE, DRE on two ASMs and DRE on three or more ASMs. A separate binary logistic regression was conducted to assess the relationship between the use of valproic acid (VPA) (yes/no) and biomarker levels, while adjusting for age, sex and mono/polytherapy. Values below the limit of quantification for GFAP were replaced with the lowest quantifiable value (50.8 pg/mL). As even minimal haemolysis can impact NSE concentration in the blood, hemolyzed samples were excluded for analyses of NSE.

## Results

### Cohort description

Among the 444 participants, the majority had focal epilepsy (62.6%) with a median epilepsy duration of 12 years (range: 0–73 years) as shown in Supplementary Table 1. The median duration of epilepsy was longer for DRE patients (17 years, range: 1–69) compared to MCE patients (12 years, range: 1–72 years).

### Association of marker levels with demographic and clinical variables in the total cohort

#### Correlation analyses

All markers were correlated with age, with NfL (*r*_s_ = 0.724, *P* < 0.001) showing a strong positive correlation. NfL was also moderately correlated with GFAP (*r*_s_ = 0.584, *P* < 0.001) and weakly with tau (*r*_s_ = −0.139, *P* = 0.003) and NSE (*r*_s_ = 0.161, *P* = 0.002). GFAP was weakly correlated with NSE (*r*_s_ = 0.108, *P* = 0.035), while S100B did not correlate with any marker. To evaluate whether the duration of the epilepsy affected marker levels beyond age, we performed a partial correlation analysis, adjusting for age. However, we found no correlations between any of the markers and epilepsy duration.

#### Clinical variables

We were then interested to see whether any of the blood markers were associated with certain clinical variables using multiple linear regressions in the whole cohort (Supplementary Tables 2–6). All models controlled for age and sex. When assessing clinical characteristics individually, polytherapy was associated with 11.1% higher values of NfL compared to monotherapy (*P* = 0.037, Supplementary Table 2) and higher S100B levels (*B*_rel_: 11.6%, *P* = 0.031, Supplementary Table 6). Higher NfL (*B*_rel_: 16.0%, *P* = 0.024) and GFAP (*B*_rel_: 38.7%, *P* < 0.001, Supplementary Table 3) were associated with acquired lesions (versus no lesion or abnormal/unrelated imaging). To investigate the joint effect of all clinical variables, we combined all variables in a model for each marker (Supplementary Tables 2–6). Having acquired lesions remained a significant predictor of GFAP (*B*_rel_: 32.6%, *P* < 0.001, Supplementary Table 3), while polytherapy also remained significantly associated with NfL (*B*_rel_: 15.7%, *P* = 0.018, Supplementary Table 2).

### Drug-resistant epilepsy

**Figure 2:**
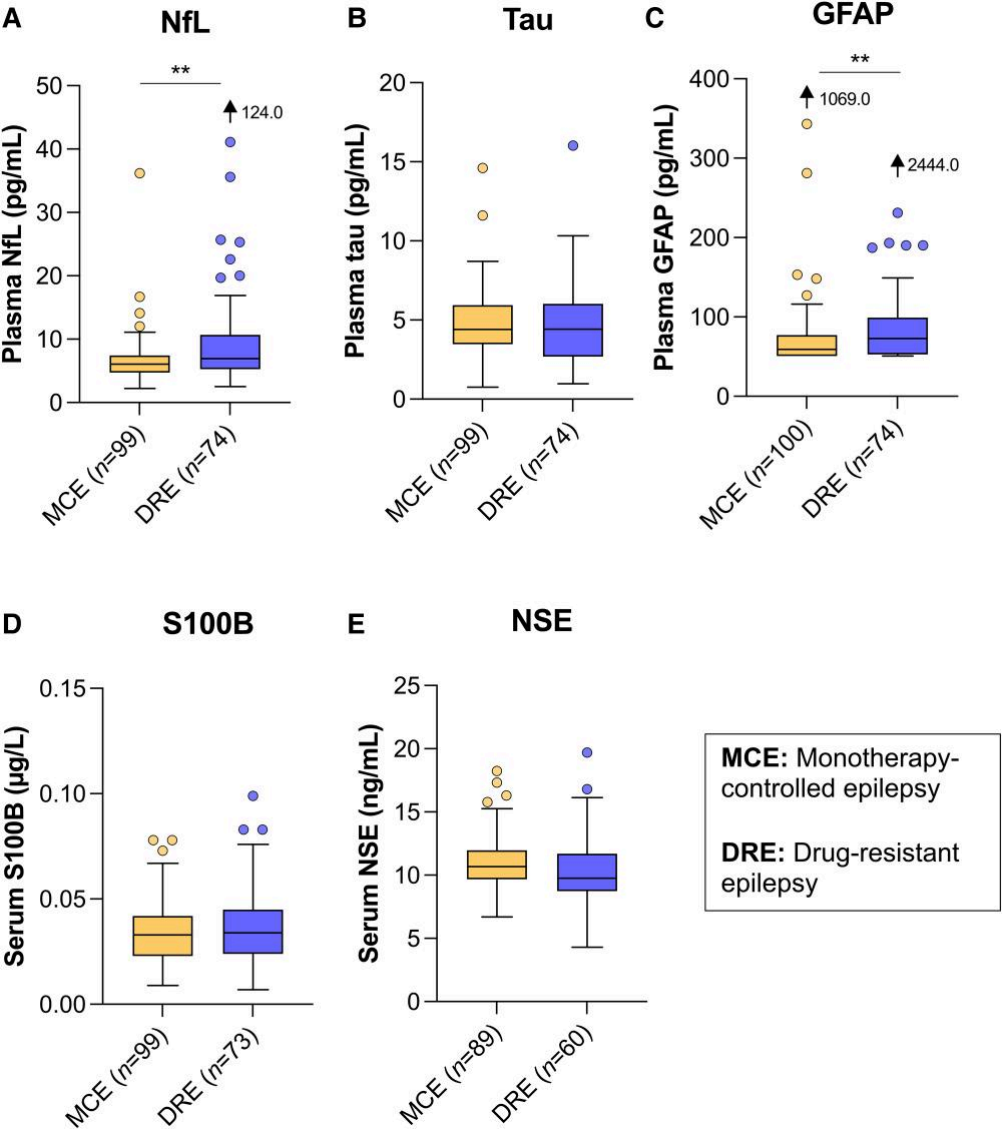
**Tukey box and whisker plot showing the distribution of marker levels in patients ≤50 years of age with DRE and MCE.** The centre line in the box represents the median value, while the upper and lower boxes represent the 75th and 25th percentiles, respectively. Whiskers extend out to 1.5 times the interquartile range from the upper and lower quartiles. (**A**) NfL (*P* = 0.002) and (**C**) GFAP (*P* = 0.006) showed significant differences in concentrations between patient groups in a Mann–Whitney U-test (two-sided, *P* < 0.05), while no significant differences were observed for (**B**) Tau, (**D**) S100B and (**E**) NSE.

**Figure 3:**
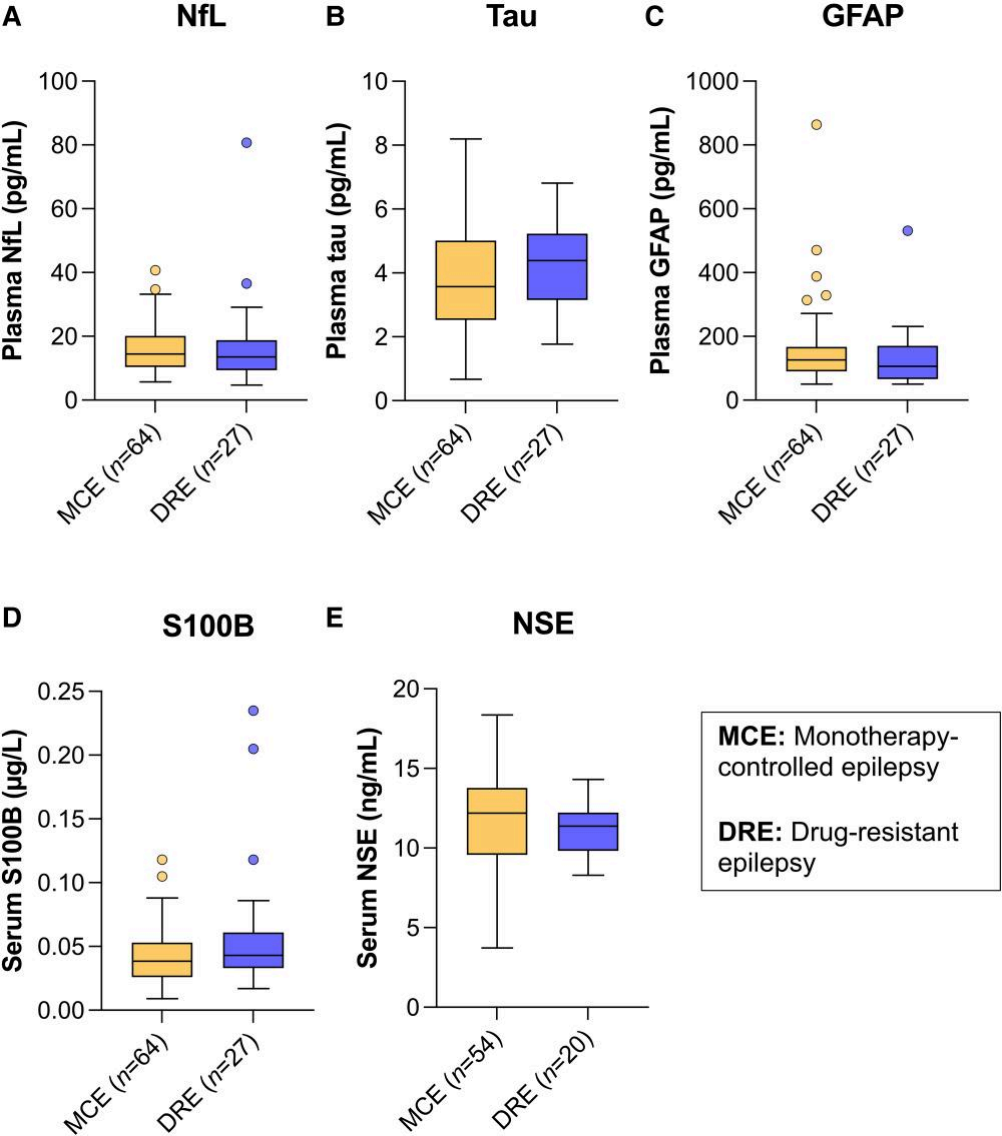
**Tukey box and whisker plot showing the distribution of marker levels in patients >50 years of age with DRE and MCE.** The centre line in the box represents the median value, while the upper and lower boxes represent the 75th and 25th percentiles, respectively. Whiskers extend out to 1.5 times the interquartile range from the upper and lower quartiles. (**A**) NfL, (**B**) Tau, (**C**) GFAP, (**D**) S100B and (**E**) NSE did not show significant differences in concentrations between patient groups in a Mann–Whitney U-test (two-sided, *P* < 0.05).

We finally compared individuals with DRE and MCE. As participants with DRE had a higher median epilepsy duration (Supplementary Table 1), we first ran separate partial correlations in patients with DRE and MCE, controlling for age. The markers did not correlate with epilepsy duration in either patient group. Because all five markers correlated with age, we compared DRE to MCE in two age groups. In participants ≤50 years of age, levels of NfL (median: 6.92 versus 6.05 pg/mL, *P* = 0.002) and GFAP (median: 72.7 versus 59.3 pg/mL, *P* = 0.006) were significantly increased in participants with DRE (Fig. 2, Table 1). None of the markers significantly differed in participants >50 years (Fig. 3, Table 1).

**Table 1:** Biomarker levels in participants ≤50 and >50 years for MCE and DRE

	≤50 years			>50 years		
	MCE	DRE	*P*	MCE	DRE	*P*
NfL (pg/mL)	(*n* = 99)	(*n* = 74)		(*n* = 64)	(*n* = 27)	
	6.05 (2.23–36.2)	6.92 (2.54–124)	**0**.**002**	14.5 (5.69–40.7)	13.5 (4.73–80.7)	0.52
GFAP (pg/mL)	(*n* = 100)	(*n* = 74)		(*n* = 64)	(*n* = 27)	
	59.3 (50.8–1069)	72.7 (50.8–2444)	**0**.**006**	126 (50.8–864)	106 (50.8–531)	0.22
Tau (pg/mL)	(*n* = 99)	(*n* = 74)		(*n* = 64)	(*n* = 27)	
	4.40 (0.75–14.6)	4.40 (0.96–16.0)	0.59	3.57 (0.67–8.20)	4.39 (1.77–6.81)	0.31
NSE (ng/mL)	(*n* = 89)	(*n* = 60)		(*n* = 54)	(*n* = 20)	
	10.7 (6.69–18.2)	9.77 (4.31–19.7)	0.054	12.2 (3.72–18.4)	11.4 (8.28–14.3)	0.11
S100B (µg/L)	(*n* = 99)	(*n* = 73)		(*n* = 64)	(*n* = 27)	
	0.033 (0.009–0.078)	0.034 (0.007–0.099)	0.74	0.039 (0.009–0.118)	0.043 (0.017–0.235)	0.20

Biomarker levels are expressed as median (range). The Mann–Whitney U-test was used for group comparisons (two-sided test, *P* < 0.05). In younger participants, significant differences were found between individuals with MCE and DRE for NfL (*P* = 0.002) and GFAP (*P* = 0.006). Bold values indicate statistical significance.

MCE, monotherapy-controlled epilepsy; DRE, drug-resistant epilepsy.

To investigate whether there is an association between higher marker levels and epilepsy status (DRE versus MCE) we ran a binomial logistic regression model for each marker while controlling for age, sex and epilepsy duration (Model 1, Table 2). NfL demonstrated a significant odds ratio of 1.06 (95% CI: 1.01–1.11, *P* = 0.022), indicating that NfL was associated with 6% higher odds for DRE per unit of NfL. When further adjusting the models for epilepsy type and structural lesions (Model 2, Table 2), the association between NfL and risk of DRE remained (OR: 1.07, 95%CI: 1.02–1.13, *P* = 0.008).

**Table 2:** Binary logistic regression analysis to assess the association between marker levels and epilepsy status

	Model 1	Model 2
	Odds ratio (95% CI), *P*-value	Odds ratio (95% CI), *P*-value
NfL	*n* = 232	*n* = 232
	**1.06 (1.01–1.11), *P* = 0.022**	**1.07 (1.02–1.13), *P* = 0.008**
GFAP	*n* = 233	*n* = 233
	1.00 (0.999–1.00), *P* = 0.67	1.00 (0.998–1.00), *P* = 0.98
Tau	*n* = 232	*n* = 232
	1.01 (0.88–1.15), *P* = 0.93	1.02 (0.88–1.19), *P* = 0.75
S100B^a^	*n* = 232	*n* = 232
	1.11 (1.00–1.25), *P* = 0.058	1.09 (0.97–1.24), *P* = 0.15
NSE	*n* = 203	*n* = 203
	0.89 (0.78–1.01), *P* = 0.066	0.90 (0.78–1.02), *P* = 0.10

Model 1: controlled for age, sex and epilepsy duration.

Model 2: controlled for age, sex, epilepsy duration, epilepsy type and lesions.

^a^Odds ratios correspond to a 0.01 µg/L increase in the original S100B protein level.

Bold values indicate statistical significance.

### Anti-seizure medications

Based on the results from the multiple linear regression analysis (Supplementary Tables 2–6), the levels of some of the markers may be confounded by the polytherapy in the DRE group. To assess this, we compared marker levels across three groups: individuals with MCE (*n* = 164), individuals with DRE on two ASMs (*n* = 54) and DRE on three or more ASMs (*n* = 47). In participants ≤50 years, both NfL (median: 8.07 versus 6.05 pg/mL, *P* = 0.006, adjusted *P*-value (*q*) = 0.012) and GFAP (median: 82.2 versus 59.3 pg/mL, *P* = 0.003, *q* = 0.006) remained significantly increased when comparing DRE on three or more ASMs (NfL *n* = 33, GFAP *n* = 33) to MCE (NfL *n* = 99, GFAP *n* = 100). NfL was also increased when comparing only individuals with DRE on two ASMs (*n* = 41) versus MCE (*n* = 99) (median: 6.76 versus 6.05 pg/mL, *P* = 0.034, *q* = 0.035). No differences were seen between individuals with DRE on two versus three or more ASMs, or in older (>50 years) individuals.

Certain medications may also impact marker levels, in particular VPA, which has been linked to brain atrophy in some cases.^36^ In our cohort, 37 individuals were currently on VPA. To evaluate whether marker levels were affected by VPA use, we ran an additional logistic regression on the total cohort, with VPA use as the binary outcome variable, adjusted for age, sex and mono/polytherapy. Here, we only found higher NSE levels to be associated with VPA (OR: 1.18, 95% CI: 1.03–1.35, *P* = 0.019). As a slightly higher percentage of individuals with DRE (13/101, 12.9%) were currently on VPA than those with MCE (11/164, 6.7%), we excluded patients on VPA and re-ran the group comparison analysis (DRE versus MCE) for each marker. Both NfL (*P* = 0.031) and GFAP (*P* = 0.018) remained significantly increased in the DRE group (≤50 years), indicating the use of VPA may not be influencing the higher levels seen in DRE in our cohort.

### Lesions

To ensure that the differences seen for NfL and GFAP did not reflect structural abnormalities related to the epilepsy, we excluded participants with lesions (see Supplementary Table 1). Levels of NfL remained significantly elevated in DRE individuals ≤50 years (*n* = 42) versus MCE (*n* = 77) (median: 6.72 versus 5.89 pg/mL, *P* = 0.029); however, the difference previously seen for GFAP was no longer significant. Similarly, when assessing marker levels only in individuals with lesions, only NfL levels remained increased in those ≤50 years (*P* = 0.034) with DRE (*n* = 32) compared to MCE (*n* = 22).

### Sensitivity analyses

When restricting the analysis to individuals with focal epilepsy, only NfL was significant in younger individuals with DRE (*n* = 59) versus MCE (*n* = 46) (median: 6.76 versus 5.62 pg/mL, *P* = 0.016). No differences were found in younger participants with generalized epilepsy. Due to the small number of patients >50 years with generalized epilepsy, we did not compare participants with generalized epilepsy in this age group.

## Discussion

DRE has been associated with neurodegenerative processes as well as cognitive deterioration, though the underlying mechanisms remain to be fully elucidated. The possibility of detecting these changes on a molecular level would benefit in a clinical setting to monitor disease progression and the development of comorbidities. Several markers known to reflect brain injury have been well-studied in various neurological conditions, such as NfL, though there is limited research in the context of epilepsy. We explored blood levels of NfL, GFAP, total tau, S100B and NSE in a regional epilepsy cohort, with a focus on DRE. We found higher levels of NfL and GFAP in participants ≤50 years with DRE compared to MCE. Furthermore, NfL levels remained significant when excluding patients with known structural brain lesions. Higher NfL was also associated with DRE in a logistic regression model after adjusting for potential confounding variables.

Elevated levels of serum NfL have previously been reported in DRE versus MCE and healthy controls.^20^ The study observed that a small number of patients with DRE exhibited abnormally high NfL levels, similar to our previous study where we also found abnormal plasma NfL in a subset of patients with epilepsy.^20,22^ In both studies, the potential effects of injury such as trauma or stroke, were controlled for suggesting the changes seen in NfL may reflect a neurodegenerative process or brain injury directly related to the drug-resistant status of the epilepsy. We also found that levels of plasma NfL remained significant after the exclusion of participants with structural lesions in the current study. Unlike NfL, the increased levels of GFAP seen in our cohort seem to reflect structural pathology rather than the seizures or chronic state of epilepsy in individuals with DRE. GFAP is considered a promising marker of clinical severity after TBI, with research indicating that plasma GFAP levels are sensitive to subclinical intracranial injuries post-TBI not detectable on CT scans.^37,38^ We cannot exclude the possibility that the higher levels of NfL we report may also indicate more subtle lesions or changes not revealed by the imaging work-up in our cohort. Additionally, changes in marker concentrations could reflect adaptive neural plasticity, such as network reorganization.

The use of multiple medications may influence the levels of NfL and GFAP. Cognitive side effects from medication have been shown to increase with the number of ASMs prescribed in polytherapy.^39^ Our results indicate that some of the markers, particularly GFAP, may be influenced to some extent by ASM frequency. While NfL appears less affected by higher ASMs in the DRE group, the relationship between ASMs and marker concentrations is likely more complex and dependent on the type of ASMs. Our analyses may not fully capture all the nuances of this interaction. Future studies should also, therefore, take into account the effects of individual and combined ASMs on marker levels in DRE.

The study has limitations due to its cross-sectional nature; longitudinal analysis would help to determine whether the changes observed in NfL and GFAP reflect a short-term increase after recent seizure activity or sustained increases indicating ongoing neurodegeneration or injury in DRE. A neuropsychological assessment would complement the biomarker findings and offer a more comprehensive understanding of the underlying associations between elevated marker levels and cognitive decline in our cohort. We also do not have information on other comorbidities or medications that may have influenced the levels of the biomarkers. It is important to mention that while ultrasensitive assays, like Simoa, provide high precision for the detection of subtle changes in biomarker levels, the clinical utility of such small changes is not clear. For instance, in the case of NfL, we observe significant yet relatively minor elevations in the DRE group. This suggests that absolute thresholds for recent seizures are unlikely to be definable. It is, however, possible that individual variations in NfL can be informative regarding seizure status, at least in a subset of patients. Repeated measures of NfL will be an important next step for understanding how the dynamics of this particular biomarker relate to seizure status.

We did not observe any significant differences in concentration for tau, S100B and NSE. The lack of observed differences may be attributed to the distinct characteristics of each biomarker that were not entirely reflected in this study. Sampling at multiple time points would, therefore, enhance our understanding of the temporal trends of these markers in epilepsy. Additionally, our cohort is relatively heterogeneous, and certain markers may only represent specific subsets of patients with particular epileptic conditions or characteristics.

Our research shows that DRE is associated with higher levels of plasma NfL and GFAP. NfL seems to be less dependent on underlying structural pathology than GFAP. The practical application of brain injury markers is limited by their elevation in a variety of CNS conditions, with some also elevated outside of CNS disorders.^40-43^ This lack of specificity indicates that the biomarkers are hardly stand-alone epilepsy tests. That does not refute clinical utility if the biomarkers are interpreted in an epilepsy context. As discussed above, future research should aim to track NfL levels over time to determine if increases are temporary or persistent in DRE cases, while accounting for more specific clinical factors such as seizure type. The wide range of CNS diseases that NfL is increased in could also be a clinical advantage. Other than after seizures, higher levels of NfL have been associated with faster disease progression in several neurodegenerative disorders and could perhaps be used as a tool to monitor disease progression in epilepsy, such as progression into a drug-resistant state or ongoing brain damage/neurodegeneration.^44-46^

## Supplementary Material

fcaf108_Supplementary_Data

## Data Availability

Results of the study contain sensitive personal data (according to Swedish research regulations) and cannot be shared by the authors.
