# Supplementary material for: Higher plasma neurofilament-light chain concentration in drug-resistant epilepsy
Source: Brain Commun. 2025 Mar 11;7(2):fcaf108. doi: 10.1093/braincomms/fcaf108 (PMC11925020; doi:10.1093/braincomms/fcaf108)
Supplement: fcaf108_Supplementary_Data [file fcaf108_supplementary_data.docx]

**Supplementary Table 1:** Demographics and clinical characteristics of study participants.

|  | **Epilepsy status** | | |  |
| --- | --- | --- | --- | --- |
|  | **Monotherapy-controlled (*n=*164*)*** | **Drug-resistant (*n*=101)** | **Undetermined (*n*=179)** | **All (*n*=444)** |
| **Age** | | |  |  |
| Median (range) | 43 years (18-86) | 41 years (18-84) | 47 years (18-92) | 44 (18-92) |
| **Gender** | | |  |  |
| Male (%) | 70 (42.7) | 47 (46.5) | 95 (53.1) | 212 (47.7) |
| Female (%) | 94 (57.3) | 54 (53.5) | 84 (46.9) | 232 (52.3) |
| **Days since last seizure (last seizure to last clinic visit or sampling)** | | |  |  |
| Median (range) | 1876 (367 – 16,497) | 11 (0 – 360) | 101 (0 – 4991) | 451 (0 – 16,497) |
| **Epilepsy duration** | | |  |  |
| Median (range) | 12 years (1 – 72) | 17 years (1 – 69) | 9 years (0 – 73) | 12 years (0 – 73) |
| **Epilepsy type** | | |  |  |
| Focal (%) | 86 (52.4) | 83 (82.2) | 109 (60.9) | 278 (62.6) |
| Generalized (%) | 36 (22.0) | 13 (12.9) | 26 (14.5) | 75 (16.9) |
| Unknown (%) | 42 (25.6) | 5 (5.0) | 44 (24.6) | 91 (20.5) |
| **Structural cause of epilepsy** | | |  |  |
| Acquired lesion (%) | 30 (18.3) | 21 (20.8) | 25 (14.0) | 76 (17.1) |
| Stroke | 11 (6.7) | 9 (8.9) | 16 (8.9) | 36 (8.1) |
| Intracerebral hemorrhage (ICH) | 8 (4.9) | 5 (5.0) | 8 (4.5) | 21 (4.7) |
| Infarction | 3 (1.8) | 4 (4.0) | 8 (4.5) | 15 (3.9) |
| Tumor | 6 (3.7) | 3 (3.0) | 3 (1.7) | 12 (2.7) |
| Trauma | 9 (5.5) | 4 (4.0) | 6 (3.9) | 19 (4.3) |
| Infection | 2 (1.2) | 2 (2.0) | 1 (0.6) | 5 (0.9) |
| Other^a^ | 2 (1.2) | 3 (3.0) | 1 (0.6) | 6 (1.4) |
| Non-acquired/other epileptogenic lesion^b^ (%) | 15 (9.1) | 30 (29.7) | 24 (13.4) | 69 (15.5) |
| No lesion or abnormal/unrelated imaging (%) | 119 (72.6) | 50 (49.5) | 130 (72.6) | 299 (67.3) |
| **Current number of anti-seizure medication** | | |  |  |
| 0 (%) | 0 | 0 | 5 (2.8) | 5 (1.1) |
| 1 (%) | 164 (100) | 0 | 122 (68.2) | 286 (64.4) |
| 2 (%) | 0 | 54 (53.5) | 39 (21.8) | 93 (20.9) |
| 3 (%) | 0 | 37 (36.6) | 11 (6.1) | 48 (10.8) |
| 4 (%) | 0 | 8 (7.9) | 1 (0.6) | 9 (2.0) |
| 5 (%) | 0 | 2 (2.0) | 1 (0.6) | 3 (0.7) |

^a^ Surgery, immune-mediated. ^b^e.g. Dysplasia, mesial sclerosis, polymicrogyria.

**Supplementary Table 2: Multiple linear regression analysis of natural log-transformed NfL and clinical variables.**

| **NfL** |  |  |
| --- | --- | --- |
|  | **Individual models** | **Combined model (*n*=352)** |
| **Predictors** | ***B*_rel(%)_ (lower, upper 95%CI), *p*** | ***B*_rel(%)_ (lower, upper 95%CI), *p*** |
| **Epilepsy type (*n*=443)** |  |  |
| Focal | . | . |
| Generalized | 3.46 (-9.43, 18.3), *p*=0.611 | 9.7 (-5.45, 27.3), *p=*0.220 |
| Unknown/cryptogenic | 1.71 (-9.79, 14.6), *p*=0.785 | 13.5 (-0.896, 30.2), *p=*0.067 |
| **Lesion (*n=*443)** |  |  |
| No lesion/unrelated | . |  |
| Acquired structural lesion | 16.0 (1.92, 31.8), *p*=0.024 | 15.6 (-0.20, 33.9), *p=*0.053 |
| Non-acquired/other epileptogenic lesion | 3.15 (-9.52, 17.5), *p=*0.645 | -0.499 (-15.0, 16.5), *p*=0.954 |
| **Days since last seizure (*n*=385)** | -0.002 (-0.004, -4.32E-6), *p*=0.05 | -0.001 (-0.004, 0.001), *p*=0.305 |
| **Epilepsy duration (*n=*391)** | -0.10 (-0.40, 0.20), *p=*0.455 | -0.2 (-0.499, 0.2), *p*=0.399 |
| **Anti-seizure medication (*n*=443)** |  |  |
| Monotherapy | . | . |
| Polytherapy | 11.1 (0.602, 22.5), *p=*0.037 | 15.7 (2.53, 30.5), *p*=0.018 |

All models are adjusted for age and sex.

*B*_rel(%)_ describes the regression coefficient (B) as a relative change in percent (*B*_rel(%)_ = (exp(b) – 1) × 100%)

**Supplementary Table 3: Multiple linear regression analysis of natural log-transformed GFAP and clinical variables.**

| **GFAP** |  |  |
| --- | --- | --- |
|  | **Individual model** | **Combined model (*n*=353)** |
| **Predictors** | ***B*_rel(%)_ (lower, upper 95%CI), *p*** | ***B*_rel(%)_ (lower, upper 95%CI), *p*** |
| **Epilepsy type (*n*=444)** |  |  |
| Focal | . | . |
| Generalized | -9.88 (-20.6, 2.33), *p*=0.109 | -4.78 (-18.2, 11.0), *p=*0.530 |
| Unknown/cryptogenic | -9.24 (-19.02, 1.82), *p*=0.097 | -1.49 (-14.4, 13.3), *p=*0.833 |
| **Lesion (*n=*444)** |  |  |
| No lesion/unrelated | . |  |
| Acquired structural lesion | 38.7 (22.9, 56.5) *p<*0.001 | 32.6 (14.0, 54.3), *p<*0.001 |
| Non-acquired/other epileptogenic lesion | 6.93 (-5.45, 20.9), *p=*0.283 | 0.904 (-14.2, 18.6), *p*=0.913 |
| **Days since last seizure (*n*=386)** | -0.002 (-0.004, 2.42E-5), *p*=0.053 | -0.001 (-0.004, 0.002), *p*=0.486 |
| **Epilepsy duration (*n=*392)** | -0.20 (-0.60, 0.10), *p=*0.125 | -0.10 (-0.499, 0.30), *p*=0.577 |
| **Anti-seizure medication (*n*=439)** |  |  |
| Monotherapy | . | . |
| Polytherapy | 6.61 (-3.05, 17.2), *p*=0.188 | 3.77 (-8.33, 17.6), *p*=0.554 |

All models are adjusted for age and sex.

*B*_rel(%)_ describes the regression coefficient (B) as a relative change in percent (*B*_rel(%)_ = (exp(b) – 1) × 100%)

**Supplementary Table 4:** **Multiple linear regression analysis of natural log-transformed tau and clinical variables.**

| **Tau** |  |  |
| --- | --- | --- |
|  | **Individual model** | **Combined model (*n*=352)** |
| **Predictors** | ***B*_rel(%)_ (lower, upper 95%CI), *p*** | ***B*_rel(%)_ (lower, upper 95%CI), *p*** |
| **Epilepsy type (*n*=442)** |  |  |
| Focal | . | . |
| Generalized | -0.499 (-12.5, 13.1), *p*=0.935 | -6.20 (-19.7, 9.75), *p=*0.425 |
| Unknown/cryptogenic | -2.66 (-13.3, 9.20), *p*=0.644 | -4.69 (-17.5, 10.2), *p=*0.515 |
| **Lesion (*n=*442)** |  |  |
| No lesion/unrelated | . |  |
| Acquired structural lesion | -4.31 (-15.6, 8.55) *p=*0.494 | -12.3 (-25.0, 2.63), *p=*0.101 |
| Non-acquired/other epileptogenic lesion | 2.22 (-9.88, 16.1), *p=*0.728 | -0.896 (-16.1, 17.0), *p*=0.914 |
| **Days since last seizure (*n=*384)** | 4.24E-4 (-0.002, 0.003), *p*=0.695 | 0.002 (-0.001, 0.004), *p*=0.244 |
| **Epilepsy duration (*n=*390)** | -0.10 (-0.399, 0.30), *p=*0.728 | -0.1 (-0.499, 0.30), *p*=0.556 |
| **Anti-seizure medication (*n*=438)** |  |  |
| Monotherapy | . | . |
| Polytherapy | 1.92 (-7.41, 12.1), *p*=0.70 | 4.39 (-8.15, 18.5), *p*=0.512 |

All models are adjusted for age and sex.

*B*_rel(%)_ describes the regression coefficient (B) as a relative change in percent (*B*_rel(%)_ = (exp(b) – 1) × 100%)

**Supplementary Table 5: Multiple linear regression analysis of natural log-transformed NSE and clinical variables.**

| **NSE** |  |  |
| --- | --- | --- |
|  | **Individual model** | **Combined model (*n*=311)** |
| **Predictors** | ***B*_rel(%)_ (lower, upper 95%CI), *p*** | ***B*_rel(%)_ (lower, upper 95%CI), *p*** |
| **Epilepsy type (*n*=383)** |  |  |
| Focal | . | . |
| Generalized | 4.19 (-2.27, 11.0), *p*=0.207 | 1.31 (-6.39, 9.64), *p=*0.744 |
| Unknown/cryptogenic | 4.19 (-1.49, 10.3), *p*=0.153 | 2.33 (-4.88, 10.2), *p=*0.532 |
| **Lesion (*n=*383)** |  |  |
| No lesion/unrelated | . |  |
| Acquired structural lesion | -5.82 (-11.4, 0.10) *p=*0.053 | -3.92 (-11.2, 3.98), *p=*0.317 |
| Non-acquired/other epileptogenic lesion | -4.21 (-9.97, 1.92), *p=*0.173 | -1.78 (-9.34, 6.50), *p*=0.666 |
| **Days since last seizure (*n=*338)** | 0.001 (-4.0E-4, 0.002), *p=*0.212 | 1.0E-4 (-0.001, 0.001), *p*=0.874 |
| **Epilepsy duration (*n=*345)** | -0.131 (-0.025 0.286), *p=*0.1 | 0.10 (-0.10, 0.40), *p*=0.155 |
| **Anti-seizure medication (*n*=378)** |  |  |
| Monotherapy | . | . |
| Polytherapy | -2.86 (-7.41, 1.92), *p*=0.235 | -3.05 (-9.15, 3.46), *p*=0.347 |

All models are adjusted for age and sex.

*B*_rel(%)_ describes the regression coefficient (B) as a relative change in percent (*B*_rel(%)_ = (exp(b) – 1) × 100%)

**Supplementary Table 6: Multiple linear regression analysis of natural log-transformed S100B and clinical variables.**

| **S100B** |  |  |
| --- | --- | --- |
|  | **Individual model** | **Combined model (*n*=350)** |
| **Predictors** | ***B*_rel(%)_ (lower, upper 95%CI), *p*** | ***B*_rel(%)_ (lower, upper 95%CI), *p*** |
| **Epilepsy type (*n*=440)** |  |  |
| Focal | . | . |
| Generalized | 6.29 (-7.23, 21.7), *p*=0.378 | 7.04 (-9.06, 25.9), *p=*0.412 |
| Unknown/cryptogenic | -2.47 (-13.6, 10.1), *p*=0.684 | -0.90 (-13.1, 17.1), *p=*0.905 |
| **Lesion (*n=*440)** |  |  |
| No lesion/unrelated | . |  |
| Acquired structural lesion | 2.53 (-10.1, 16.9) *p=*0.708 | 2.02 (-13.2, 19.7), *p=*0.810 |
| Non-acquired/other epileptogenic lesion | 2.84 (-10.1, 17.5), *p=*0.684 | 2.63 (-13.6, 21.8), *p*=0.769 |
| **Days since last seizure (*n=*382)** | 4.0E-4 (-0.002, 0.003), *p=*0.726 | -2.0E-4 (-0.003, 0.003), *p*=0.901 |
| **Epilepsy duration (*n=*389)** | 0.269 (-0.0561, 0.595), *p*=0.105 | 0.10 (-0.30, 0.602), *p*=0.498 |
| **Anti-seizure medication (*n*=435)** |  |  |
| Monotherapy | . | . |
| Polytherapy | 11.6 (1.01, 23.4), *p*=0.031 | 10.6 (-3.05, 26.1), *p*=0.133 |

All models are adjusted for age and sex.

*B*_rel(%)_ describes the regression coefficient (B) as a relative change in percent (*B*_rel(%)_ = (exp(b) – 1) × 100%)
